# A COMMUNITY-ENGAGED RESEARCH JOURNEY: USING HOME REPAIRS TO PROMOTE HEALTH EQUITY AMONG OLDER ADULTS

**DOI:** 10.1093/geroni/igad104.0755

**Published:** 2023-12-21

**Authors:** Sarah Filec, Gail Schechter, Mike Laz, Rachel O’Conor

**Affiliations:** Feinberg School of Medicine, Northwestern University, Chicago, Illinois, United States; Housing Opportunities & Maintenance for the Elderly, Chicago, Illinois, United States; Housing Opportunities & Maintenance for the Elderly, Chicago, Illinois, United States; Northwestern University, Chicago, Illinois, United States

## Abstract

Homeownership is a primary mechanism to build wealth. The majority of older Chicagoans own their home, but stark racial inequities exist due to discriminatory lending and purchasing practices. Homeownership requires regular maintenance, but low-income older adults are often forced to defer maintenance for financial reasons, which can lead to hazardous living environments, falls, and the need to move. Intuitively, housing conditions play a pivotal role in enabling older adults to continue to live in their home safely, yet few studies have examined the independent benefit of home repairs towards preserving homeownership and improving older adults’ health. In 2019, stakeholders from community based organizations (CBO) and academic researchers convened a partnership identifying shared research goals and priorities focused on the impact of housing support services on older adult health. Over the past four years, we formalized a partnership and through iterative conversations with experts in aging and housing, and older adults with lived experience, our team identified a need to evaluate home repairs and their impact on 1) health outcomes, and 2) preserved homeownership and independent living for older adults. Our team iteratively co-developed a pilot study, with all stakeholders (older homeowners, CBOs, researchers) informing the methodology and research questions. In January 2023, we collectively launched our research pilot to evaluate the impact of a home repair program on health outcomes and homeownership preservation among predominantly Black low-income older adults in Chicago. Together we established a trusted partnership that will continue to address root causes of health inequities of older adults.

